# The aplication of *Pistacia khinjuk* extract nanoemulsion in a biopolymeric coating to improve the shelf life extension of sunflower oil

**DOI:** 10.1002/fsn3.2057

**Published:** 2020-12-08

**Authors:** Marziehalsadat Hosseinialhashemi, Javad Tavakoli, Alireza Rafati, Fatemeh Ahmadi

**Affiliations:** ^1^ Department of Food Science and Technology Faculty of Agriculture & Nutrition, Sarvestan Branch Islamic Azad University Sarvestan Fars Iran; ^2^ Department of Food Science and Technology Faculty of Agriculture Jahrom University Jahrom Fars Iran; ^3^ Division of Pharmaceutical Chemistry and Food Science Sarvestan Branch Islamic Azad University Sarvestan Fars Iran; ^4^ Department of Pharmaceutics School of Pharmacy Shiraz University of Medical Sciences Shiraz Fars Iran

**Keywords:** antioxidant activity, *Balango*, *Fenugreek*, nanoencapsulation, oxidation

## Abstract

In the present study, a hydroalcoholic extract of *P. khinjuk* was obtained by sonication method at 60°C for 50 min. The measurement revealed that the total phenolic content of the extract was 46.0 mg/g. The results showed that the extract has an antioxidant activity of 73.5% and 8.3 (µmol TE/g DW) in DPPH radical scavenging method and FRAP assay, respectively. Also, Balango (*Lallemantia royleana*) and Fenugreek (*Trigonella foenum‐graecum*) seed gum and their composition (1:1) were used to prepare the nanoemulsion with *P. khinjuk* extract. The droplet mean size of nanoemulsions was ranged from 310.34 to 354.19 nm. The highest encapsulation efficiency was observed in Balango nanoemulsion. *P. khinjuk* extract nanoemulsion coating with Balango and TBHQ was added to sunflower oil at 200 and 100 ppm, respectively. During 24‐day storage at 60°C, samples were investigated for peroxide, acid, and *p*‐anisidine values at 4‐day intervals. The results showed that oils containing nanoemulsion had the highest stability during storage. However, in all samples peroxide, acid and *p*‐anisidine values increased but the rate of oxidation in samples containing both synthetic and natural antioxidants was slower than the control sample.

## Graphical abstract

In the present study, Balango (Lallemantia royleana) and Fenugreek (Trigonella foenum‐graecum) seed gum and their composition (1:1) were used to prepare the nanoemulsion with P. khinjuk extract. The highest encapsulation efficiency was observed in Balango nanoemulsion.

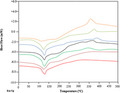

## INTRODUCTION

Vegetable oils are known as essential compounds of the human diet due to their nutritional and medicinal values. Sunflower oil is rich in polyunsaturated fatty acids (PUFA) (linoleic acid, 59%) and monounsaturated fatty acids (MUFA) (oleic acid, 30%). The high susceptibility of sunflower oil to lipid oxidation is also attributed to its high PUFA content. (Chong et al., 2015). Oil oxidation involves unfavorable reactions in edible oils and oil‐containing foods and often is regarded as the main cause of shelf life reduction and deterioration of oils quality, affecting color, flavor, and texture and also has adverse effects on health (Chong et al., 2015; Mahjoob et al., 2018; Rafiee et al., 2017). Thus, the utilization of antioxidants is the most appropriate way to dominate the stability problems associated with fats and oils. Also, it could be considered as a good option to prevent the oxidation of oils and increase their stability during storage. Synthetic antioxidants such as tert‐butyl hydroquinone (TBHQ), butylated hydroxylanisole (BHA), and butylated hydroxyl toluene (BHT) have shown carcinogenic and mutagenic effects in human (Tavakoli et al., 2017).

Recently, great attention has been directed to the field of natural antioxidants in order to find natural compounds with various health‐promoting effects (Chong et al., 2015; Kazemi et al., 2020; Tavakoli et al., 2017). Some investigations demonstrated the antioxidant effects of *Pistacia* fruit family. It has been shown that the presence of phenolic compounds is responsible for high antioxidant capacities of fruit extracts (Pedraza‐Chaverri et al., 2008).

*Pistacia khinjuk* (Anacardiaceae) is one of the *Pistacia* species widely distributed in Iran and is named Kolkhoung in the Persian language. This plant has various biological activities and is used for different pharmacological activities for instant antidiabetic, antitumor, anti‐cholinesterase, antimicrobial, and antifungal activities (Taran et al., 2010). The application of *P. khinjuk as a natural compound in* traditional Persian medicine has been reported in several studies. This applications include the prevention and treatment of motion sickness, stomach discomfort, nausea, and vomiting such as motion sickness, stomach discomfort, nausea, and vomiting, (Dob et al., 2006). The *P*. *khinjuk* fruit extract is also used in pharmaceutical industry. Kolkhoung extract majorly contains flavonoids and phenolic compounds. It is reported that its fruits and resins have strong antioxidant activity due to being rich in secondary compounds (Hatamnia et al., 2016).

Moreover, direct use of phenolic compounds is not applicable due to unpleasant taste, interaction with food components, and potential decomposition reactions These decomposition reactions take place during the processing of food and their storage due to the different conditions including light and oxygen as well as temperature and enzymes. The encapsulation of extract is a successful strategy to overcome the concerns by improving the physicochemical stability of extract and protecting it from interactions with food ingredients (Fang & Bhandari, 2010). Encapsulation technology could be defined as the process of packaging materials in solid or liquid state in the small capsules enabling their release in a controlled rate manner. Various different materials could be used as encapsulating compounds including proteins and hydrocolloids. in both food and pharmaceutical industries (Anbinder et al., 2011; Esfahani et al., 2020; Mousavian et al., ). Due to the popular demand of natural biopolymers, the incorporation of phenolic compounds in natural wall materials as a value‐added component is a growing area of research. Balango seed (*Lallemantia royleana*) is a plant with mucilaginous property which could be found in Asia, Europe, and the Middle East. The extracted Balango seed gum (BSG) is a high molecular weight gum with a slightly flexible chain (Salehi et al., 2014). Fenugreek (*Trigonella foenum‐graecum* ) is a leguminous plant grown in the Mediterranean, northern Africa, western Asia (Brummer et al., 2003), and Iran. Fenugreek seed gum (FSG) has been used medicinally and as a condiment for many years. Recently, research interest has increased about seed components (Roberts et al., 2012). The choice of appropriate material for encapsulation has shown crucial role in the process of encapsulation. Bioactive materials could be encapsulated using various synthetic polymers with the food‐grade quality. However, investigators are always seeking for cheap, abundant, natural, and popular biopolymers. Some studies have applied seed gums to encapsulate food ingredient for instant beet dye, in the combination of chia seed mucilage, maltodextrin, and Arabic gum (Antigo et al., 2020), bergamot essential oil in Balango hydrocolloids (Rezaeinia et al., 2019), and anthocyanins of black raspberry encapsulated based on fenugreek gum (Yousefi et al., 2015).

Up to date, there is no study indicating the antioxidant capacity of *P. khinjuk* extract in the nanoemulsion form for improving the storage time of sunflower oil. The aim of the present investigation was the evaluation of antioxidant activity of *P. khinjuk* extract nanoemulsion on the oxidative stability of sunflower oil. Therefore, first, the effect of type of seed gum, *Balango* and *Fenugreek* seed gum, on the characterization of nanoemulsion was studied, and second, the antioxidant activity of the free and encapsulated extract in sunflower oil was measured.

## MATERIALS AND METHODS

### Materials

Fresh *P*. *khinjuk* fruits were collected in October from the Meimand city (Fars, Iran, summer of 2019). Balango gum and fenugreek gum seeds were purchased from Tabibdaru Company in Shiraz City (summer of 2019). Narges Shiraz Oil Co. kindly provided refined, bleached, and deodorized sunflower oil without antioxidants. All other reagents were of analytical grade and purchased from Sigma‐Aldrich (St. Louis, MO, USA).

### Preparation of *P. khinjuk* Extract

First, 50‐gram powder of *P. khinjuk* fruit was dispersed in 250 ml of ethanol/water (60:40) solvent. Then the Erlenmeyer flask of the samples was placed in the ultrasonic bath (DT 102H; BANDELIN) (35 kHz, for 50 min at 65°C) (Estakhr et al., 2020).

### Determination of DPPH radical scavenging activity and FRAP test

Evaluation of DPPH radical scavenging assay and ferric reducing antioxidant power (FRAP) test was done using by method described by Tavakoli et al. (2019).

### Seed gum extraction

Seed gums of Balango and Fenugreek were extracted using reported method by Salehi et al. (2014). The cleaned Balangu and Fenugreek seeds were soaked in distilled water (water/seed ratio 20:1) at 50°C, pH = 7, for 20 min. Separation of the hydrocolloid from the swollen seeds was achieved by passing the seeds through an extractor equipped with a rotating plate that scraped the gum layer on the seed surface. The extracted solution was then filtered and dried in an air forced oven at 50°C (convection oven, Memmert Universal, Schwabach, Germany), and finally, the powder was milled, packed, and kept at cool and dry condition.

### Preparation of biopolymer solutions and Nanoemulsions

Biopolymer solutions of Balango and Fenugreek gums and complex of Balango gum and Fenugreek gum (1:1) were prepared using the explained method by Estakhr et al. (2020) and Delfanian et al. (2018).

Balango and Fenugreek seed gums were mixed in deionized water to achieve a total solids content of 0.5% (w/w). A magnetic mixer was used to the better dissolution of the compounds for 15 min at 20 Cº, and then, the solutions were kept in the refrigerator for 24 hr. The complex solutions of gums were prepared by adding a Balango gum solution into the solution of Fenugreek gum and stirring at 20 Cº. Tween 80 as emulsifier was added to the gum solution at 2% w/w concentration. The P. *khinjuk* extract (200 ppm) was added to the mixture at a ratio of 1:5, and after half an hour stirring, the ultraturrax homogenizer (Ultraturrax T25, Janke & Kunkel, Germany) at 12,000 rpm and 21,000 rpm was used for homogenization during 5 min. Then, to further reduce the particle size, the probe type ultrasonic (PRO‐250, mLabs, USA) with six cycles and 30 s was used. Nanoemulsions were frozen for 24 hr at −20°C and then by using a freeze‐drying machine at 0.017 mPa and −57°C for 48 hr were dried (Chranioti et al., 2016).

### Encapsulation efficiency and total phenolic content

Encapsulation efficiency of nanoencapsulated powder was done using method described by Robert et al. (2010). In order to measuring the total phenolic content of different extract and oil samples was used the described method by Delfanian et al. (2018).

### Characterization of nanoemulsion

Droplet mean size, polydispersity index (PDI), and zeta potential of the *P.khinjuk* extract‐containing nanoemulsion were analyzed using the described method by Harris et al. (2011).

### Surface Electronic Morphology of Nanoemulsion

The shape and surface of different nanoemulsions were observed by scanning electron microscopy (*SEM*). The samples were examined using a scanning electron microscope (JEOL JSM‐6400, JEOL, Tokyo, Japan).

## DSC

To evaluate the thermal properties of nanocapsules, the thermal analysis was performed by differential scanning calorimeter device (DSC 821e, Mettler Toledo, Germany). 5 mg of each nanocapsule was accurately sealed in a DSC aluminum pan and heated from 10 to 500°C with a constant rate of 10°C/min under the nitrogen flow rate of 20 ml/min. The energy (mW) given to the sample was determined and further evaluations were performed (Amiri et al., 2020).

## FTIR

To confirm the formation of the nanoencapsulated *P. khinjuk* extract, the Fourier transformation infrared (FTIR) spectrum was tested using a spectrometer (Equinox 55, Bruker, Germany) at 25°C. The measurements were performed with a resolution of 4 cm^−1^ at the range of 1000–4000 cm^−1^ (Zou et al., 2006).

### Release properties

The release properties were determined according to Mohammadi et al. (2016). The stability of nano‐coatings was measured based on the release of phenolic compounds from nanoemulsions.

### Acid value, peroxide value and p‐anisidine value

The determination of Acid Value, peroxide value and p‐anisidine value of the different oil samples was done based on the method ascribed by Tavakoli et al. (2017), Kazemi et al. (2020) and Estakhr et al. (2020), respectively.

### Peroxide value and p‐anisidine value

The determination of peroxide value and p‐anisidine value of the different oil samples was done based on the method ascribed by Kazemi et al. (2020) and Estakhr et al. (2020), respectively.

### Statistical analysis

The mean ± *SD* values obtained from experiments were subjected to analysis of variance by performing one‐way ANOVA test. Duncan test was performed to determine the significant differences between means and the *p* values <.05 were considered as significant.

## RESULTS AND DISCUSSION

### Total Phenolic Content and Antioxidant Activity

Medicinal plants that contain secondary metabolites have been known for antioxidant activities (Adrar et al., 2016), and they can be used as food preservatives (Bakry et al., 2016). In the present study, *P. khinjuk* extract was investigated for total phenolic content and antioxidant activity and data are depicted in Table 1. The total phenolic content of *P. khinjuk* was 46.0 mg/g DW. Antioxidant activity of extracts is one of the biological properties of great interest because they may preserve food from oxidation and spoilage reactions (Miguel, 2010). *P*. *khinjuk* extract (200 ppm) showed 73.5% DPPH radical scavenging and 8.3 (µmol TE/g) ferric reduction activities. Various plant extracts have shown antioxidant activity and, in many cases, this activity is secondary to the presence of active components, mainly attributable to phenolic compounds. Our result was close to the report of a study performed by Ahmed et al. (2017) and Shojaei et al. (2011) for *P.khinjuk* extract who reported *P*. *khinjuk* extract shows antioxidant activity due to presence of phenolic content and have approved our results.

**TABLE 1:** The antioxidant activity of *P. khinjuk* extract and its phenolic content

Sample (ppm)	Total phenolic content (mg/g DW)	DPPH (%)	FRAP (µmol TE/g)
*P*.*khinjuk* (200)	46.0 ± 0.0	73.5 ± 2.3^a^	8.3 ± 0.6^a^
TBHQ (100)	‐	68.17 ± 4.5^b^	7.7 ± 0.8^b^

Note

Mean ± *SD* within a column with the same lowercase letters are not significantly different at *p* < .05.

### Encapsulation efficiency and nanoemulsion characteristics

Encapsulation efficiency is an important parameter to be taken into attention when developing an encapsulation process. The nanocapsules obtained showed a relatively high value of encapsulation efficiency (over 50%). The nanocapsules with the *Balango* seed gum wall showed the highest encapsulation efficiency. It seems that interaction between gums caused a decrease in phenol: gum bound encapsulation efficiency.

Droplet size could be considered as a determining parameter in stability and encapsulation efficiency. Table 2 demonstrates that the size of all the samples is in the nanometer range which is a promising feature for their further applications. No difference (*p* > .05) in particle size was observed between nanocapsules with a single wall meaning the type of gum did not affect this parameter. The difference of the zeta potential among the samples was statistically significant (*p* < .05). The net charge of anionic groups in gums and carboxyl groups of *P. khinjuk* extract involves in the negative zeta potential of nanocapsules. The best PDI was achieved at nanocapsules with a composite wall. In other words, the lower PDI represents the smaller size of nanocapsules. Presumably, when the particle distribution index increased, the nanocapsules are more likely to aggregate together, so the particle size incremented.

**TABLE 2:** Characteristics of different nanoemulsions

Coating	Encapsulation Efficiency (%)	Particle Size (nm)	PDI	Zeta potential (mv)
*Fenugreek*	60.64^b^	354.19^a^	0.46^a^	−11.25^c^
Composite	59.06^b^	310.34^c^	0.37^c^	−17.36^a^
*Balango*	63.46^a^	337.08^ab^	0.41^b^	−14.37^b^

Note

Mean ± *SD* within a column with the same lowercase letters are not significantly different at *p* < .05. *Fenugreek: P*. *khinjuk* extract coated with *Fenugreek* seed gum*, Balango: P*. *khinjuk* extract coated with *Balango* seed gum, and Composite*: P*. *khinjuk* extract coated with *Fenugreek* and *Balango* seed gum (1:1)

### Surface Electronic Morphology of Nanoemulsion

**FIGURE 1:**
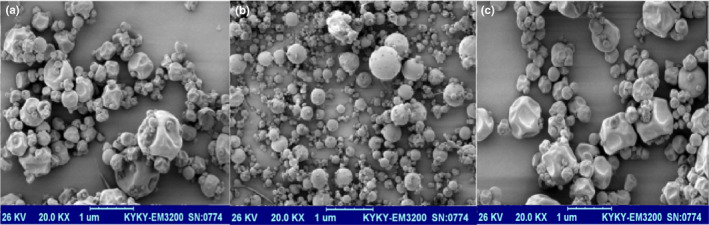
*SEM* images of *P.khinjuk* extract nanoemulsion in different coatings. a: *Fenugreek,* b: composite, c: *Balango*

Figure 1. shows the morphology of *P. khinjuk* extract nanoemulsion. All nanoemulsions prepared were spherical in shape. Nanoemulsion with a composite coating showed uniform and round structure and it would be attributed to the potential interactions between polysaccharide groups of *Fenugreek* and *Balango* gum. Two influential factors on the morphology of capsules are bioactive components and wall materials. Nanocapsules with a single‐layer wall showed the larger size and the results of particle size confirmed this observation.

### Differential Scanning Calorimetry (DSC)

**FIGURE 2:**
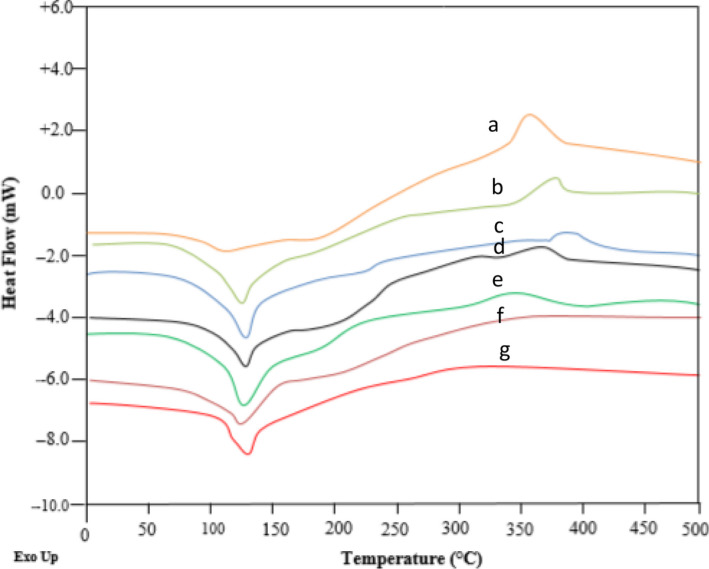
DSC thermograms of encapsulated *P*.*khinjuk* extract a: *P*.*khinjuk* extract, b: fenugreek seed gum/ *P*.*khinjuk* extract, c: composite seed gum/ *P*.*khinjuk* extract, d: Balango seed gum/*P*.*khinjuk* extract, e: fenugreek seed gum, f: composite seed gum, g: Balango seed gum

Figure 2 represents the thermal analysis data of *P. khinjuk* extract after and before encapsulation. These data have obtained from DSC analysis and indicated that the encapsulation using seed gum materials was carried out successfully. The evaporation pint of the extract was observed at 357°C. This peak is associated with the melting endotherm peak of *P. khinjuk* extract. The presence of *P. khinjuk* extract affects the thermograms. The melting behavior of the encapsulated samples could be significantly affected by the addition of *P. khinjuk* extract. Also, transition temperatures were increased in encapsulated *P.khinjuk* extract and occur in higher temperatures. It indicates the elevation of thermal resistance of *P. khinjuk* extract in nanocapsules. Similar results are reported by Musuc et al. (2013) who found that rosemary extract changed the melting behavior of extract loaded in biopolymer foil. The broader peaks with lower intensity were observed for the encapsulated samples containing *P. khinjuk compared with the pure* extract samples. It seems that the changes in crystallite size distribution result in the broadening of the melting peaks following the encapsulation of the extract. The results demonstrated that the extract was entrapped in the network formed by the seed gum (Musuc et al. 2013). This resulted in the improvement of its thermal resistance which is a determining parameter for the application of encapsulated extract in food industry.

## FTIR

**FIGURE 3:**
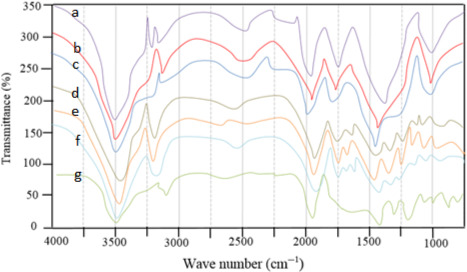
FTIR spectra of a: Balango seed gum, b: fenugreek seed gum, c: composite seed gum, d: balango seed gum/*P*.*khinjuk* extract, e: fenugreek seed gum/ *P*.*khinjuk* extract, f: composite seed gum/ *P*.*khinjuk* extract, g: *P*.*khinjuk* extract

FTIR spectra of *P. khinjuk* extract, seed gums powder, and CSO nanoparticle have been presented in Figure 3. FTIR analysis is performed to investigate the interaction between gums as a wall material and the extract. FTIR analysis can provide useful information about shape changes, chemical structure, maximum IR absorption intensity, and sample peaks. As can be seen, the study area in this study is in the range of 1,000 to 4,000 (cm^‐1^). Generally, the specific peaks between 3,200 and 3,500 cm^−1^ show the presence of –OH and ‐NH stretching in *P. khinjuk* extract and fenugreek seed gum/*P.khinjuk* extract. The broad peak at the range 3100–3250 cm^−1^ exhibited the presence of ‐CH stretching (alkyne and alkene) and hydroxyl group in nanocapsules. Also, the bands at 2250–2750 cm^−1^ associated with the O = C=O stretching which was observed at seed gums composite and separately and related to hydroxyl groups and because of the existence of –OH in phenolic compounds can be said that *P. khinjuk* extract certainly encapsulated by seed gum. Rezaei Erami et al. (2019) reported fairly similar peaks in encapsulated *bitter gourd* extract.

### Release properties

**FIGURE 4:**
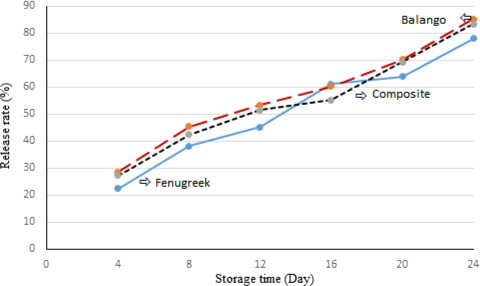
Release trend of *P.khinjuk* extract from nanocapsule with different wall materials for 24 days at 60 ◦C. *Fenugreek: P*. *khinjuk* extract coated with *Fenugreek* seed gum*, Balango: P*. *khinjuk* extract coated with *Balango* seed gum, and composite*: P*. *khinjuk* extract coated with *Fenugreek* and *Balango* seed gum

The encapsulation of bioactive materials is carried out in order to improve their release profile in a controlled manner. In this investigation, the impact of wall materials on the release of *P. khinjuk* phenolic compounds from nanocapsules during 24 days storage at 60 ◦C was evaluated by measuring the total phenolic content (Figure 4). After 12 days, approximately 50% of total phenolics were released. The highest release index was observed in nanocapsule with a balango seed gum coating. This could be attributed to the phenolic compounds' bounds and outer layer of aqueous phases. While extract coated with Fenugreek seed gum released just about 77.96% of their phenolic compounds at the end of storage time, which was significantly (*p* < .05) slower than extract coated with composition of two seed gums. These findings obviously demonstrated that a complex of seed gums has controlled the release of phenolic compounds during storage. Generally, the release of phenolic compounds has been affected by the characteristics of the layers formed around the extract. At the first storage time, composite nanocapsule with the least particle size displayed the faster phenolic compound release. It seems that the rupture of wall materials in the nanocapsules led to the decrease of droplet size of the nanocapsules. The results are consistent with the other investigations including Mohammadi et al. (2016) and Estévez et al. (2019) in which the release rate of olive leave phenolic compounds and grape seed phenolic‐rich extract were increased during their storage. According to the release properties of phenolic compounds, the best sample was nanoemulsion created with balango seed gum, which was selected to investigate the effect of nanoemulsion extract on oxidation of sunflower oil.

### Oil oxidation

#### Peroxide value

**FIGURE 5:**
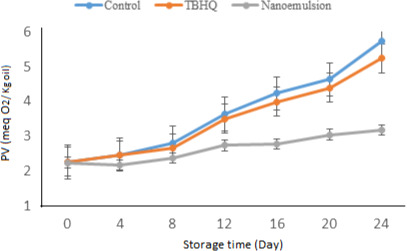
Change in peroxide value of different samples during storage time. Nanoemulsion: nanoemulsion created with balango seed gum

The degree of primary oxidation of sunflower oil (SFO) was evaluated using the measurement of PV of oils containing nanoemulsion and TBHQ and oil without antioxidants at 60°C for 24 days. As demonstrated in Figure 5, all treated samples showed an increase in their PVs during the storage. This could be the effect of the formation of lipid hydroperoxides. (Iqbal & Bhanger, 2007). The lipid oxidation results in the formation of lipid hydroperoxidases. These hydroperoxides are able to induce the degradation of the oil by decomposition into nonvolatile and volatile secondary products. This could be used as an index for the initiation of oxidation (O’Keefe & Pike, 2010). PV was in the range 2.24–3.17 meq/kg for oil‐containing nanoemulsion, while it was 5.24 mEq/kg for the sample with TBHQ after storage up to day 24 (Figure 5). The highest value of PV for the control samples was achieved after 24 days of storage which was 5.73 mEq/kg. The highest PV was observed for the control sample at all stages, followed by SFO containing 100 ppm of TBHQ. Higher PV values were recorded for the control samples in comparison with the antioxidant and synthetic antioxidants, demonstrating that nanoemulsion extract acted efficiently to reduce lipid oxidation.

The metal chelating and free radical scavenging activities of plant phenolic compounds could be considered as the major reasons for their effect in reducing the lipid oxidation (Maqsood et al., 2014). The decay of lipid hydroperoxides by phenolic compounds leads to the delay in lipid oxidation process. There are various phenolic compounds showing different efficiencies in the retardation of the lipid oxidation (Maqsood et al., 2013). These results are consistent with the observations reported by Delfanian et al. (2018), who used nanoencapsulated Bene hull polyphenols to increase the oxidative stability of soybean oil. In another study, the nanoliposomes were prepared to load pistachio green hull extract and the final formulation was added to soybean oil. The results of this study demonstrated that 500 and 700 mg of free and encapsulated extracts were more effective for retarding oxidation of soybean oil in comparison with control samples (Roostaee et al., 2017).

#### Acid value

The acid value could be considered as an index showing the quality of oil during the storage at accelerated condition. Table 3 depicts the impact of antioxidants (natural and synthetic materials) on the acid values of sunflower oils after 24 days of storage at accelerated condition (i.e., 60°C). This increase can be attributed to the hydrolysis of triglycerides and the production of carbonyl groups during the autoxidation process (Sayari et al., 2015). Generally, in all samples, AV dramatically increased. These results are consistent with the observations reported by Sayari et al., (2015) who reported AV of sunflower oil increased during the storage period and oil without antioxidant exhibited the highest AV. The lower acid value represents the higher stability of the oil samples. Also, the acid values of all the samples demonstrated significant differences at various storage periods (*p* < .05). This difference was not observed for the samples stored between 0 and 4 days. The sunflower oil containing the nanoemulsion formulation of the extract demonstrated lower acid values compared with the same oil‐containing TBHQ (*p* < .05).

**TABLE 3:** Change in acid value of different samples during storage (mg KOH/g)

Sample	0 day	4 day	8 day	12 day	16 day	20 day	24 day
Control	0.16 ± 0.0^Fa^	0.26 ± 0.0^Fa^	0.45 ± 0.0^Ea^	0.63 ± 0.1^Da^	0.81 ± 0.0^Ca^	0.99 ± 0.1^Ba^	1.18 ± 0.1^Aa^
TBHQ	0.19 ± 0.0^Ea^	0.19 ± 0.0^Eb^	0.27 ± 0.0^Db^	0.34 ± 0.0^Cb^	0.42 ± 0.1^Bb^	0.45 ± 0.1^ABb^	0.50 ± 0.0^Ab^
Nanoemulsion	0.18 ± 0.0^Ea^	0.18 ± 0.0^Eb^	0.22 ± 0.0^Dc^	0.26 ± 0.0^Cc^	0.29 ± 0.1^BCc^	0.31 ± 0.2^ABc^	0.34 ± 0.1^Ac^

Note

Mean ± *SD* within a column with the same lowercase letters are not significantly different at *p* < .05. Mean ± *SD* within a row with the same uppercase letters are not significantly different at *p* < .05. Nanoemulsion: nanoemulsion created with balango seed gum

#### *p*‐Anisidine Value

Once the hydroperoxide breaks down to aldehydes, carbonyl, and ketones, the secondary lipid oxidation product is produced. This conversion could be measured by the *p*‐anisidine (*p*‐AnV) value. This stage leads to the rancid flavor of oil The better quality of oils is significantly associated with the lower *p*‐AnV value at the primary stages of their storage. (O’Keefe and Pike, 2010). According to our results, all samples demonstrated higher p‐anisidine values during their storage. (Table 4). The pattern of the increase of *p*‐AnV under the acceleration condition after 24 days of storage showed that the highest *p*‐AnV value belongs to SFO while the TBHQ and nanoemulsions showed lower values. The results also showed that all *p*‐AnVs for each treatment were significantly different at different storage times. This difference was not observed between the days 0 and 4. The p‐AnV value of SFO containing TBHQ was consistently higher than SFO with nanoemulsion. This reveals that nanoemulsion extract has shown promising results for the retardation of secondary oxidation products formation in sunflower oil while it is stored at 60°C compared to TBHQ, a synthetic antioxidant. There was a significant difference (*p* > .05) between the p‐anisidine value of samples containing TBHQ and nanoemulsion. This could be the results of less effectiveness of TBHQ in the inhibition of the formation of secondary oxidation products. Compared with the natural antioxidant materials, our results are consistent with previously published results such as the work by Delfanian et al. (2018), which reported nanoencapsulated Bene hull polyphenols controlled oil oxidation in comparison with control samples.

**TABLE 4:** Change in anisidine value of different samples during storage

p‐anisidine	0 day	4 day	8 day	12 day	16 day	20 day	24 day
SFO	3.54 ± 0.1^Ea^	3.83 ± 0.1^DEa^	4.01 ± 0.1^Da^	4.34 ± 0.1^Ca^	4.52 ± 0.0^Ca^	4.78 ± 0.1^Ba^	5.31 ± 0.1^Aa^
TBHQ	3.54 ± 0.0^Ea^	3.63 ± 0.1^Eb^	3.97 ± 0.1^Da^	4.17 ± 0.0^Cb^	4.35 ± 0.1^BCb^	4.53 ± 0.1^Bb^	5.23 ± 0.0^Aa^
Nanoemulsion	3.5 ± 0.0^Ea^	3.53 ± 0.0^Eb^	3.60 ± 0.0^DEb^	3.71 ± 0.0^CDc^	3.72 ± 0.1^Cc^	4.08 ± 0.2^Bc^	4.67 ± 0.1^Ab^

Note

Mean ± *SD* within a column with the same lowercase letters are not significantly different at *p* < .05. Mean ± *SD* within a row with the same uppercase letters are not significantly different at *p* < .05. Nanoemulsion: nanoemulsion created with balango seed gum

Our results showed that 200 ppm of nanoemulsion extract could sufficiently protect the formation of secondary oxidation products in comparison with the application of 100 ppm of TBHQ. Hence, *P. khinjuk* extract nanoemulsion can be recommended as an alternative source of antioxidant to prolong the shelf life of sunflower oil.

## CONCLUSIONS

The studies to date on *P.khinjuk* extract nanoemulsion suggested that because of the presence of phenolics compounds, it can be used as an important natural antioxidant source. *P. khinjuk* extract has higher antioxidant activity than TBHQ in free and encapsulated form. It is important to consider that the *P*. *khinjuk* extract nanoemulsion demonstrated sufficiently high antioxidant activity in sunflower oil. In conclusion, the oil‐containing P. *khinjuk* extract nanoemulsion was found to possess good oxidation stability, and based on our findings, it seems that it could be considered as a promising alternative for synthetic antioxidants in the food industry.
